# Quantitative Data Quality Assurance, Analysis and Presentation

**DOI:** 10.1111/jpm.13143

**Published:** 2024-12-19

**Authors:** Paul Slater, Felicity Hasson

**Affiliations:** ^1^ Institute of Nursing and Health Research Ulster University Antrim UK

**Keywords:** data analysis, data management, quantitative

## Abstract

Quantitative data quality assurance is the systematic process and procedures used to ensure the accuracy, consistency, reliability, and integrity of data throughout the research process. Effective quality assurance helps identify and correct errors, reduce biases, and ensure the data meets the standards needed for analysis and reporting. This paper provides an overview of key issues to consider when working with data and reporting findings.

## 1 Introduction

Quantitative data are broadly defined as any data that are measured using numerical values. It helps identify patterns, trends and look for relationships and differences between variables through objective and verifiable measurement and statistical testing. This paper examines the preparatory work involved in the managing a dataset prior to full statistical analysis.

**FIGURE 1:**
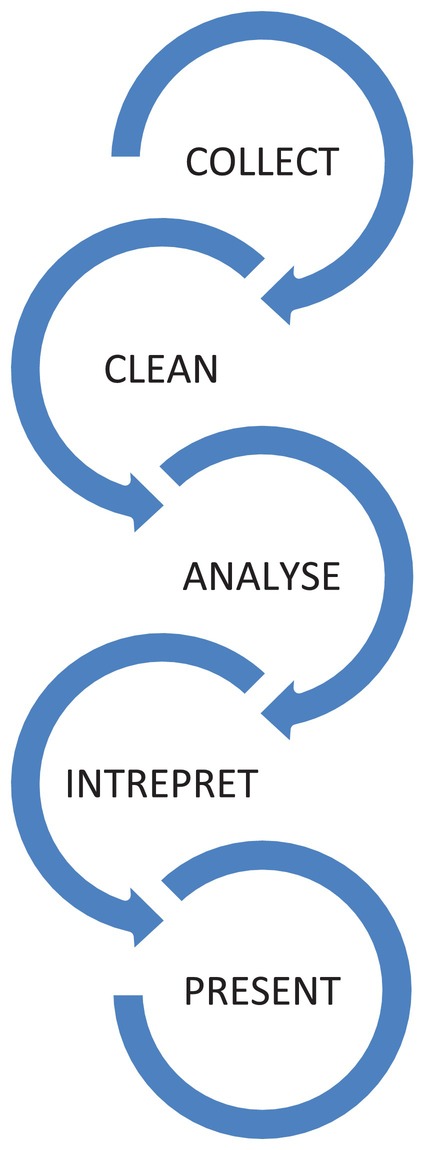
The stages of quantitative data management model.

Once you have defined your study design, variables of interest and measurement type (see Slater and Hasson 2024a), the data management process follows a rigours step‐by‐step process (see Figure 1). Each stage is equally as important as the next and requires the researcher to interact with the dataset in an iterative process to exact the relevant information in a rigorous and transparent manner. In the following five sections, we will outline key points to consider when conducting quantitative research.

## 2 Collecting the Data

Once you have established the objectives of the study, the next stage focuses on the collection of the data. Quantitative data can be collected using a variety of methods and the main types are identified in previous paper (Slater and Hasson 2024b).

## 3 Cleaning the Data

When data are collected, they are seldom at a stage that is ready for statistical analysis. The proper management of quantitative data is essential prior to analysis process, the presentation and interpretation of research findings. The researcher needs to ‘quality assure’ the data prior to full analysis. The data that you have collected require cleaning to reduce errors or inconsistencies. Doing so helps to enhance your data quality. These processes often fail to be reported in research papers; however, they form a very important part of the research process. The main issues are identified here.

- Checking the data for duplications: If the data are collected online then there is a potential that a respondent may have completed the questionnaire twice. You therefore need to identify and remove any identical copies of data, leaving only unique participant data in your dataset.
- Removal of questionnaires with certain thresholds of missing data: Missing data are a common occurrence, especially in paper‐based questionnaires; however, it is recognised as a difficult issue to address. Firstly, a distinction needs to be made regarding what constitutes missing data and not relevant data. Missing data are data that have been omitted but a response is expected: not relevant data are data that the respondent does not necessarily need to complete, for example—not applicable. In quantitative research, data are precious and we aim to retain it as much as possible. If a sample size calculation threshold has been exceeded, then the removal of incomplete questionnaires is easier to manage. If not, or no particular sample size is expected, then we try to retain as much data as possible.
  As a researcher you must decide when to remove participants and their corresponding data. This can be achieved by statistical analysis and setting percentage thresholds of completion. The percentage levels of missing data in questionnaires can be calculated using a Missing Completely at Random (Little's MCAR) test and a decision made regarding (1) establishing a threshold for inclusion/exclusion and (2) the pattern of missingness for remaining data. The MCAR test provide statistical analysis of whether missing data are truly random or is a samples' reluctance to complete a specific question, as well and providing the percentage of missing data per question and participant. There are no hard or fast rules regarding the threshold for removing incomplete participant data. It can be set at lower limits (say 50% completeness) to retain more respondents or set at 100% to maintain the integrity of the dataset. The removal of participants needs to be reported as it may be indicative of instrument fatigue (the questionnaire is too long).
  The pattern of missingness is concerned with whether missing data are truly random or if they are caused by some underlying factor. The Little's MCAR test will provide statistical evidence of this. If data are missing completely at random, then the missingness introduces no bias to the results. If the data are not missing at random, then advanced imputation methods may be required.
- If missing data are present in a dataset, then it can be managed statistically. There are a series of statistical techniques to compensate for missing data such as Missing Values Analysis, estimation maximisation or mean scores brought forward. Each have their strengths and weaknesses associated with them and should be further explored before deciding which option is most appropriate for your study design (see Baraldi and Enders 2010 for more information).
- Checking the data for anomalies: This refers to data that deviate from the expected/ usual patterns. As most real‐world datasets will have some differences from the ‘normal samples’, it is your job to detect and respond to them appropriately. This is as simple as running descriptive statistics for all measures and examining the responses to ensure that all responses are in line with expected counts. For example, all Likert scales are within the boundaries of the scoring range. This aids the identification of anomalies and correction before the full analysis.
- Summation to constructs and/or clinical definitions. Instruments or screening tools, such as the PHQ9 or the GAD7, may include instructions that permit classification of presence or severity of clinical conditions. Likewise, instruments may have Likert scales summated to a construct level. Instrument user manuals will instruct the researcher how to do this and what items to include in the analysis.

## 4 Data Analysis

This is the step that strikes the most fear into novice quantitative researchers, as quantitative data analysis requires the use of statistical methods to describe, summarise and compare data. The process tends to proceed in a series of waves of analysis, which provides the researcher with the opportunity to build upon a rigorous protocol, working up to a point where hypotheses can be tested. *It also allows the researcher to save data files as a series of steps so they can retrace their steps should they need to!*

There are two main cycles of quantitative data analysis. Descriptive analysis is used to summarise or describe the dataset; for example, frequencies, mean, median and mode. Inferential analysis is used to compare, analyse relationships or make predictions from the data, hence enabling you to draw conclusions. Various statistical methods (parametric tests vs. non‐parametric) assume that the distribution of the population data is normal distribution, therefore you must test for normality. Therefore, to help you decide the most appropriate statistical tests to undertake, you must assess the normality of the data. A normality test indicates whether your dataset stems from a normal distributed population.

### 4.1 Measures of Normality of Distribution of data

There are various methods available which can help you test the normality of data. See below the psychometric properties of the measures.

- Measures of normality of distribution are a central assumption of parametric statistical tests. Kurtosis refers to the peakedness or flatness of the distribution around the mean. Skewness refers to the deviation of data around the mean score. Values of ± 2 for both measures would indicate normality of distribution. However, with larger samples these values are more likely to be violated and become less significant. Other tests such as the Kolmogorov–Smirnov and Shapiro–Wilk tests are used to indicate normality of distribution (For fuller details please see Ghasemi and Zahediasl 2012).

Running descriptive statistics of data provides the foundation of all future analysis. It provides the researcher with the opportunity to eyeball the data—to explore what are the trends and patterns of responding in the data. The simplest way to do this is to run frequencies scores and measures of central tendency (means, standard deviations) as dictated by the data type. The analysis can be done in two runs—(1) looking at socio/demographic characteristics and (2) all other data. The socio/demographic details can form an outline of the sample.

Psychometric properties should be established prior to further analysis of any standardised instrument. Reliability and validity are the cornerstone of all research: without these properties all research is questionable (Slater, McCance, and McCormack 2017). Modern instrument development follows rigorous guidelines such as COSMIN (Mokkink et al. 2010). Statistical tests include structural validity (factor analysis) and test–retest (see Souza, Alexandre, and Guirardello 2017; Mikkonen, Tomietto, and Watson 2022). The most reported psychometric measure is a Cronbach's alpha. Cronbach's alpha test examines whether the items are measuring an underlying construct. Scores of > 0.7  are considered acceptable. For example, the generalised anxiety disorder (GAD 7; Spitzer et al. 2006) comprises of seven items measuring the construct ‘anxiety’. If you cannot report the psychometric properties for your study sample, you should at least report the psychometric properties if the tool had been used in other studies involving a similar sample.

**FIGURE 2:**
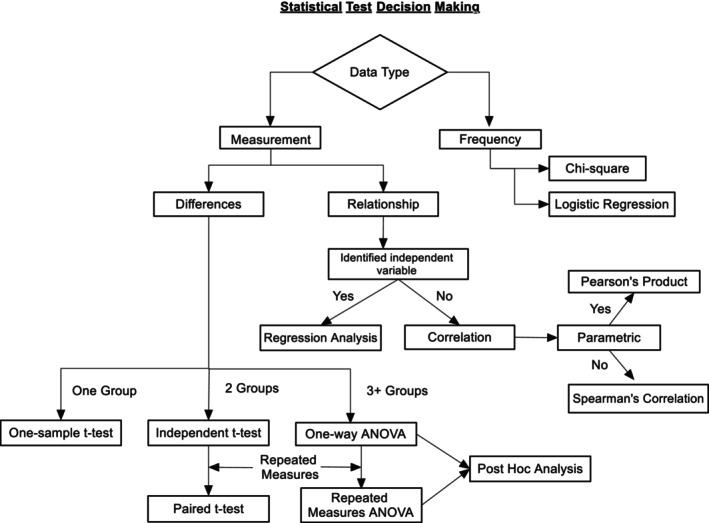
Statistical test decision‐making flow diagram.

These data are ready for further in‐depth statistical analysis. Deciding on what test to use can be daunting; however, the statistical test decision‐making flow diagram (see Figure 2) can assist in this process. Understanding the study's fundamental details such as cross‐sectional or longitudinal; measurement type—nominal, ordinal or scale; or normally distributed will help you navigate through the decision‐making tree (Figure 2). For example, if the data are nominal then chi‐squared test and logistic regression are used. If its measurement and looking at relationships, then its correlation or regression analysis, depending on whether we want to see the impact of independent variables on the scores. These details have been discussed in previous papers (Slater and Hasson 2024b). The statistical tests outlined here will be followed up in further detail in subsequent support papers.

## 5 Interpretation of Data

Writing the report and deciding what to report or not report and how to present the data appropriately needs careful consideration. For example, in peer‐reviewed publications, you will be confined by a word limit and limited to a certain number of tables/figures as outlined in the authorship guidelines. Therefore, you have careful decisions to make on what interpretations of the data you present.

The interpretation and presentation of statistical data is very important and must be conducted in a clear and transparent manner. There are several issues that must be considered.

- Avoid being selective in your reporting: At the commencement of your study, you set out clear objectives which your results should address.
- Avoid (or correct for) multiplicity: Multiplicity, or when multiple comparisons are run, increase the likelihood of a chance association or spurious interpretation of findings. When multiple tests cannot be avoided (such as in post hoc analysis), the significance threshold most be adjusted. Fortunately, post hoc analysis such as Bonferroni test automatically corrects for multiplicity.
- Report statistically significant and non‐significant finding: You need balance what you report to ensure both significant and non‐significant findings are presented. Doing so may help to prevent future research(ers) from going down unproductive rabbit holes. There is no need to further investigate non‐significant findings. For example, if a one‐way analysis of variance (ANOVA) is not significant then there is no necessity to run post hoc tests.
- Write your publication in line with journal/institute guidelines: For example, for numbers greater than 100, report to the nearest whole number (e.g.—M = 7850). For numbers between 10 and 100, report to one decimal point. For numbers between 0.10 and 10, report to two decimal points; for numbers less than 0.10, report to 3 decimal points. Report p‐values as exact values, even for non‐significant results.

## 6 Presentation of Data

The presentation of findings for consideration in a journal is an essential skill. The reality of publishing research is that you are restricted by the journal guidelines. A 5000‐word limit translates to having approximately 1250 to write up a results section. Therefore, it is essential that you capture as much information as feasible in other formats such as tables, figures and diagrams. This also helps breaks up the monotony of written text.

Tables are an extremely useful method of presenting a large volume of information in a clear and structured manner. For example, traditionally the demographic characteristics of the study sample are presented in tablature form. This allows the reader to see the sample characteristics clearly. When drawing a table or a figure, do not simply write about every detail contained in the table. Simply highlight the most interesting points in the table and be sure to direct the readers' eye to these points in the table. Be careful not to overload a table as it might become too cluttered and difficult to understand.

Graphs and figures can help to demonstrate complex issues in a visual manner, making it easy for the reader to grasp the significant issues. This is especially when describing procedural or relational pathway such as structural equational modelling. Consult journal guideline relating to tables, graphs and figures permitted and whether they contribute to the overall word count.

## 7 Conclusion

Many of the points raised in this paper are not reported in detail in the peer‐reviewed quantitative papers presented in any journal. However, they are essential components to be considered when conducting a statistical study and are accrued with experience. Hopefully this paper will help highlight these issues and direct researchers to helpful links. Remember the mantra of the academic world ‘Publish or Perish’ and share your research findings with others so they may avail of your learning.

## Ethics Statement

The authors have nothing to report.

## Conflicts of Interest

The authors declare no conflicts of interest.

## Data Availability

The authors have nothing to report.
